# Benefits of a near-peer program from the tutors’ perspective: a survey of Australian junior doctors in a regional teaching program

**DOI:** 10.1186/s12909-025-06762-2

**Published:** 2025-02-27

**Authors:** David Medveczky, Alicia Mitchell, Eleonora Leopardi, Amanda Dawson

**Affiliations:** 1 Central Coast Clinical School, Joint Medical Program, School of Medicine and Public Health, University of Newcastle, Callaghan, NSW 2308 Australia; 2 Central Coast Local Health District, New South Wales Health, Gosford, NSW 2250 Australia

**Keywords:** Near-peer teaching, Tutor perspective, Junior doctor, Teaching program

## Abstract

**Background:**

Near-peer teaching has been shown to provide diverse benefits for both tutees and tutors in senior medical student and junior trainee settings. However, junior trainees may face more obstacles in teaching including competing clinical priorities and time management. We sought to investigate the challenges and benefits of engaging in near-peer teaching for junior trainees within our local context. Our Near-Peer Medical Teaching (NPMT) teaching program is designed and facilitated by junior doctors for medical students at the Central Coast Clinical School (University of Newcastle) of the Joint Medical Program.

**Methods:**

Current and past NPMT tutors participated in an online survey from October 2022 to April 2023. Tutors were asked about feasibility of teaching within a work environment, perceived benefits from their experience and attitudes towards medical education.

**Results:**

Teaching experience appears to be influenced by competing clinical priorities and convenience of session times, but it does not appear to exert considerable stress on tutors likely due to self-selection of tutors with prior enjoyable teaching experience. Furthermore, this study indicates that junior doctors derived enjoyment and developed clinical skills and professional qualities, which are important factors in increasing job satisfaction and ameliorating burn-out in this cohort.

**Conclusions:**

Junior doctors appear to benefit from engaging in near-peer programs in the Australian teaching hospital setting. Further research should include qualitative methodologies to explore the perspectives of Australian junior doctors’ more deeply.

**Supplementary Information:**

The online version contains supplementary material available at 10.1186/s12909-025-06762-2.

## Introduction

Near-peer teaching is an educational strategy where teaching is conducted by medical trainees at least one year senior than their learners [1]. Near-peer teaching has been shown to provide benefits not only for tutees but also for tutors in both senior medical student and junior medical trainee settings. Senior medical student tutors report consolidation of taught content [2–7], development of professional attributes including communication skills, teamwork, ability to give feedback and organisational/planning skills [2, 5, 7–13] and increased desire towards engagement in medical education and academia in the future [6, 8, 11, 13, 14]. Similarly, junior doctor tutors appear to receive benefits including perceived improvements in knowledge and skills [15–19], professional qualities [17, 19, 20] and teaching ability and knowledge of educational principles [15, 18, 20, 21], as well as fostering positive attitudes towards teaching and aspirations to work in medical education [15–19, 22].

While there are multiple Australian studies exploring senior medical student tutor perspectives in near-peer programs [23, 24], data regarding the experience of junior doctors in near-peer programs are more limited [25]. Unlike senior medical students, junior doctors teach in a working environment with competing clinical responsibilities and there are ongoing concerns of junior doctor burnout—particularly in context of the COVID19 pandemic [26]. Recent UK studies have described the teaching experiences of junior doctors and have identified tensions between clinical and teaching priorities that were felt to influence teaching style and quality [27, 28]. Therefore, we aimed to explore the perspectives of junior doctor tutors within our near-peer program through a survey. Our survey addressed the following areas:

- Feasibility of teaching within a work environment
- Stress associated with teaching in a clinical environment
- Self-perceived improvements in clinical knowledge and professional qualities
- Attitudes towards teaching and aspirations to pursue opportunities in medical education

## Methods

### Study setting

The Near-Peer Medical Teaching (NPMT) teaching program of the University of Newcastle is designed and facilitated by junior doctors for medical students at the Central Coast Clinical School of the Joint Medical Program. NPMT was founded in 2016 and engages students during their clinical placements at Gosford and Wyong hospitals, Central Coast Local Health District. Interns (first post-graduate year) or residents (second post-graduate year) offer teaching sessions to medical students, engaging in one or more teaching formats of the program including bedside teaching, small-group tutorials, and simulated skills sessions. Teaching is primarily offered in the disciplines of Medicine, Surgery and Critical Care, with opportunities to engage with sub-specialty disciplines, e.g. Paediatrics, Radiology.

Every year 60–70 junior doctors are recruited to the Central Coast Local Health District, and one third to half of each cohort choose to participate in the NPMT program. Approximately 50–75 tutors are active within the program in some capacity at any one time. Within the program, junior doctors participate on a flexible basis, delivering anywhere from a single skill session to regular weekly bedside teaching. This allows for self-management of workload without a pre-established requirement of commitment. Tutors are offered teaching support and guidance via on-demand educational resources in an accessible online repository (npmteaching.com.au), featuring consultant-reviewed lesson plans and guidance regarding facilitating teaching sessions. Additionally, an annual teaching skills workshop is organised to introduce the incoming tutors to key principles of delivering teaching in the formats offered in the program, and supporting learners’ education in near-peer settings.

### Study design

This study employed a descriptive cross-sectional design to capture the perspectives of the current and past junior trainees volunteering in the program in regards to the challenges and benefits of engaging in near-peer teaching in addition to their clinical duties.

### Study sample

We asked all current and past NPMT tutors to participate in our study by completing an anonymous online survey hosted on a secure RedCAP database from October 2022 to April 2023. Tutors were contacted via email registered on the scheduling website used to book teaching sessions and via our social media Facebook group of tutors. Furthermore, tutors who answered the survey were encouraged to share the survey with their colleagues who participated in the program.

### Survey design

Data was collected via a structured survey, which was designed in line with Artino et al.’s guide to survey design within medical education [29]. Questions were generated by the research team following consideration of the existing literature investigating engagement with near-peer teaching as junior trainees, and available evidence on junior trainees’ wellbeing [2–12, 14–25, 27, 28, 30–35]. Tutors were asked to provide non-identifying demographic information, and answer questions about perceived benefits from their experience, feasibility of teaching alongside their clinical duties in the work environment and attitudes towards medical education. At the end of the survey, there was an optional free text answer box that respondents could use at their discretion to provide additional descriptions of their experience of the program.

### Data analysis

Respondents were considered to have attempted the survey if they had completed at least the first section (demographics). Only complete survey responses were included in the analysis. To evaluate responses with regards to questions about likelihood/improvements, visual analogue scales were used with labels at 0, 50 and 100. The labels at 0, 50 and 100 were varied according to each individual question but generally 0 was labelled never/no benefit, 50 was labelled sometimes/some benefit and 100 was labelled always/strong benefit. For these responses, means and 95% confidence intervals were calculated using SPSS Statistics Version 26.0. Means and percentages for other data were calculated using Excel. Optional free text answers were also integrated into the visual analogue scale data using abductive reasoning.

## Results

We received 32 responses to our survey, of which 26 were complete. Of the 26 complete responses, 6 provided optional free-text responses at the end of the survey. As we were unable to record the exact number of potential participants the survey was distributed to, we are unable to provide a precise figure for the response rate. Our best estimate is that the survey was available to 250 current and former NPMT members, with a complete response rate of approximately 10%. The survey instrument has been included (see Additional file 1).

The characteristics of the survey respondents are shown in Table 1. Most respondents were current (80.1%) rather than past tutors. Approximately 70% of respondents had less than one year of teaching experience within the program and approximately 75% were post graduate year two or less. With regards to university attendance, 2/3rds of respondents graduated from undergraduate programs, with half of all respondents being graduates of the Joint Medical Program (University of Newcastle/University of New England, Australia). Approximately 2/3rds of respondents reported engaging with a teaching activity at least once a fortnight. The most common speciality area of involvement in the program was Medicine (80.8%) followed by Surgery (53.8%).

**Table 1:** Characteristics of survey respondents (*n* = 26)

Characteristic	Mean (SD) or n (%)
**Age**	27 (2.5) years
**Female**	12 (46.2)
**Current tutors**	21 (80.8)
**Teaching duration**	
Less than 1 year	18 (69.2)
1 – 2 years	4 (15.4)
More than 2 years	4 (15.4)
**PGY level**	
PGY1	9 (34.6)
PGY2	10 (38.5)
PGY3 or above	7 (26.9)
**University attendance**	
Undergraduate	17 (65.4)
Joint Medical Program (University of Newcastle/University of New England)	13 (50)
Postgraduate	9 (34.6)
**Average teaching engagement**	
Less than once per month	4 (15.4)
Once per month	5 (19.2)
Once per fortnight	12 (46.2)
Once per week	5 (19.2)
**Specialty areas of involvement**^**a**^	
Medicine	21 (80.8)
Surgery	14 (53.8)
Critical Care (Emergency, Anaesthetics and Intensive Care)	12 (46.2)
Radiology	6 (23.1)
Obstetrics and Gynaecology	2 (7.7)
Paediatrics	1 (3.8)
Psychiatry	1 (3.8)
**Motivations for involvement**^**a**^	
Prior enjoyable teaching experience	24 (92.3)
Giving back to medical community	20 (76.9)
Adding to CV	21 (80.8)
Consolidation of own knowledge/skills	17 (65.4)
Interesting in working in medical education	17 (65.4)

^a^Categories are not mutually exclusive

Table 2 compares teaching experience gained prior to participating in the NPMT program vs experience within the program itself. The overwhelming majority of tutors reported past teaching experience prior to engaging with the NPMT program (93.7%), which is in keeping with prior enjoyable teaching experience being the most common motivation for engagement in the NPMT program. However, none of the participants reported a formal education qualification outside of teaching experience in their medical degree. Experience in bedside teaching was the most common area of involvement in the NPMT program for respondents (73.1%), followed by tutorials and skills sessions.

**Table 2:** Prior vs current teaching experience within the NPMT program (*n* = 26)

	**Prior experience, n (%)**	**NPMT experience, n (%)**
Nil	2 (7.7)	-
Bedside teaching	15 (57.7)	19 (73.1)
Tutorials	19 (73.1)	17 (65.4)
Skills sessions	12 (46.2)	16 (61.5)
Resource writing	7 (26.9)	10 (38.5)
Workshop attendance	8 (30.8)	8 (30.8)
Formal/mock examination	13 (50.0)	12 (46.2)
Leadership role	11 (42.3)	7 (26.9)

**Fig. 1:**
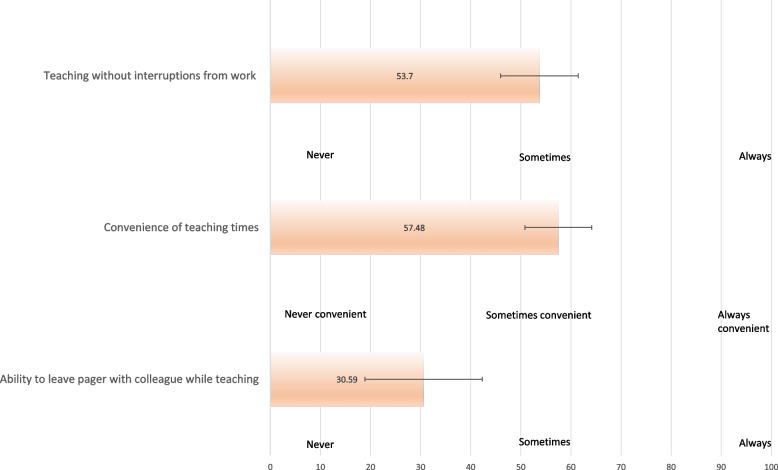
Feasibility of teaching at work (*n* = 26)

Figure 1 displays tutor responses that pertain to the feasibility of teaching in context of clinical responsibilities. Respondents indicated that they could only ‘sometimes’ teach without interruptions from work (mean 53.7, 95%CI 45.9–61.5) and teaching times were only ‘sometimes convenient’ (mean 57.5, 95%CI 50.8- 64.1, 0 = never, 50 = sometimes, 100 = always). Furthermore, tutors conveyed that they could only occasionally leave their pager with a colleague while teaching (mean 30.59, 95%CI 18.9 – 42.3, 0 = never, 50 = sometimes, 100 = always). In describing their experience, one respondent highlighted competing clinical responsibilities, particularly in medical departments, as a barrier to teaching: *‘I tend to pick up sessions when I have an ADO [allocated day off]/ on relief/ on evenings/ on ED days off. Unfortunately, I was unable to participate in teaching whilst on a busy medical term. I would love to continue teaching into residency, however given that I'm medically inclined, I suspect I will have more medical terms and will not be able to teach as much.’* These findings likely partially explain why approximately 1/3rd of respondents indicated that they undertook teaching activity solely outside working hours.

With regards to stress associated with teaching, respondents indicated that prior teaching before participating in the NPMT program was somewhat stressful (mean 31.9, 95%CI 22 – 41.8, 0 = minimally stressful, 50 = sometimes stressful, 100 = majorly stressful). Participants expressed that there was some relief of stress associated with teaching through participating in the NPMT program (mean 58.3, 95%CI 49.6–66.9, 0 = no relief, 50 = some relief, 100 = strong relief). A collegiate environment (50%) and the NPMT website (38.4%) were the main factors in relieving stress associated with teaching, with logistical support and teaching workshop attendance being ranked lower. Respondents indicated that they found the NPMT website quite useful (mean 69.7, 95%CI 60.7–78.5, 0 = not at all useful, 50 = somewhat useful, 100 = strongly useful).

On average, tutors reported to experience enjoyment (mean 83.6, 95%CI 77.5–89.6, 0 = did not enjoy at all, 100 = strongly enjoyed) and to receive somewhat strong overall benefit (mean 77.4, 95%CI 71.3–83.5, 0 = no benefit, 100 = strong benefit) from participation in the program. These positive findings were corroborated by one respondent that identified enjoyment and benefit: *‘I enjoyed my experience with NPMT this year. It has been wonderful working within other tutors and students. It helped reinforce my passion for teaching. I found it valuable for myself in reinforcing knowledge and learning. I found delivering sessions in a collegiate setting was enjoyable. I look forward to working within NPMT next year!’.*

**Fig. 2:**
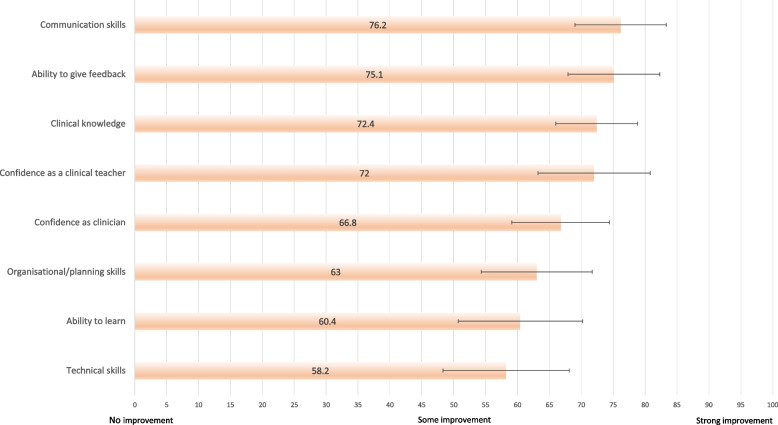
Perceived improvements in knowledge, confidence and professional qualities (*n* = 26)

Figure 2 illustrates perceived improvements in clinical knowledge, skills and professional qualities from engagement in the NPMT program. Respondents indicated the strongest self-perceived improvements in the domains of clinical knowledge (mean 72.4, 95%CI 66.0–78.8), communication (mean 76.2, 95%CI 69.0–83.3) and feedback delivery (mean 75.1, 95%CI 67.9–82.3, 0 = no improvement, 100 = strong improvement).

The weakest self-perceived improvements were seen in own ability to learn (mean 60.4, 95%CI 50.7–70.2) and technical skills (mean 58.2, 95%CI 48.3–68.1) (0 = no improvement, 100 = strong improvement).

**Fig. 3:**
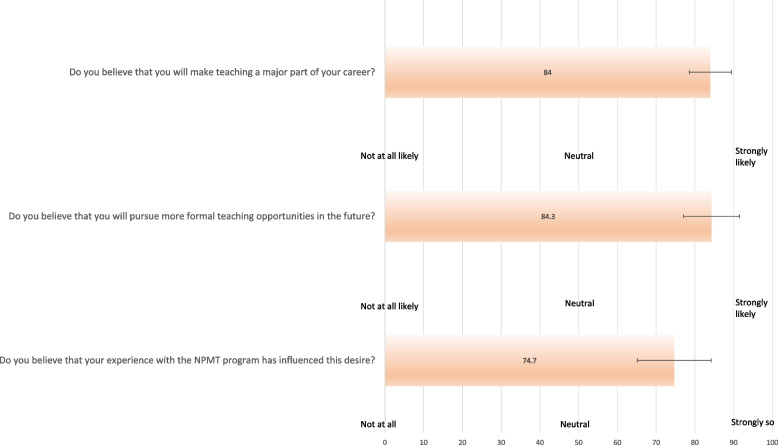
Future teaching aspirations (*n* = 26)

Figure 3 displays tutors’ responses relating to future teaching aspirations. Respondents indicated a fairly strong likelihood to make teaching a major part of their future careers (mean 84, 95%CI 78.6–89.4) and to pursue formal teaching opportunities in the future (mean 84.3, 95%CI 77.1–91.5) (0 = not at all, 50 = neutral, 100 = strongly likely). Furthermore, respondents indicated that their experience with the NPMT program has somewhat strongly influenced this desire (mean 74.7, 95%CI 65.2–84.2) (0 = not at all, 100 = strongly so).

Regarding attitudes to medical education, tutors indicated that they would somewhat strongly recommend the program to themselves if they were a student (mean 90.7, 95%CI 86.95–94.45) (0 = would not recommend at all, 100 = would strongly recommend). Respondents reported a fairly definite belief that every doctor should participate in medical education (mean 76.75, 95%CI 66.79–86.71) and expressed a semi-strong desire to continue to improve teaching skills (mean 87.4, 95%CI 81.12–93.68) (0 = not at all, 100 = strongly so). One respondent elaborated on the importance of vertical transmission of knowledge in medicine: *‘NPMT has been a useful tool to engage with medical students and enables doctors of all levels to impart our knowledge and skills to said medical students. It also tests our ability to teach others which is a difficult skill to master but is necessary the more senior you become as a clinician. Would recommend others to try at least once in teaching others to see how you can improve as a teacher.’*

Another respondent described their engagement in near-peer teaching following the NPMT experience as below: *‘I have introduced the NPMT curriculum to (other) hospitals throughout my training … At (other) hospitals, it [NPMT] has been an adjunct to PRINT [pre-intern] terms and greatly beneficial to students. We ran it in an entirely online format in the setting of COVID restrictions.’*

## Discussion

This study aimed to evaluate the experience of tutors participating in our near-peer program. Overall, we found that tutors reported diverse benefits across several domains, including deriving enjoyment from the NPMT program and perceived improvements in clinical knowledge, skills and professional qualities. We did not find substantial reduction of stress associated with teaching within the program.

There are many studies reporting similar benefits to junior doctor participation in North American ‘resident-as-teacher’ programs [16]. Furthermore, a national Australian study on near-peer teaching in general practice revealed that 68% of prevocational trainees participated in some form of near-peer teaching and trainees reported perceived improvements in their clinical skills, knowledge, and communication skills by undertaking teaching [25]. However, to our knowledge these are the first Australian data regarding a junior doctor hospital-based near-peer program. We consider that our program differs from near-peer teaching delivered in a general practice setting, as it is primarily run by junior trainees (post-graduate years 1 and 2), rather than registrars (post-graduate year 3 and beyond), who have accumulated more experience as independent clinical practitioners.

The findings that tutors were only able to sometimes teach without interruptions, found teaching times only sometimes convenient and were usually unable to leave their pager with a colleague are consistent with similar studies overseas. Competing clinical responsibilities were highlighted as problematic particularly during busy medical terms. Irish and English studies of near-peer teaching delivered by interns found similar organisational issues including difficulties with finding convenient teaching times and balancing teaching time with clinical responsibilities [27, 28]. We concur with the authors of those studies: our experience shows that high level organisational support is required for near-peer programs to improve junior doctor engagement, including negotiation with hospital administration and advocacy from resident association bodies.

Given recent concerns for junior doctor burnout, we hypothesised that teaching may be associated with high amounts of stress and that engagement with the NPMT program may mitigate this. However, respondents indicated relatively low levels of stress associated with teaching prior to participation in the program and minimal relief associated with participation. We postulate that this is due to the finding that almost all respondents reported prior enjoyable teaching experiences, and to the highly flexible commitment allowed in our program. Although not identified in this survey, we assume that most prior enjoyable teaching was likely teaching in near-peer programs during medical school. The widespread adoption of near-peer teaching into medical school curricula is likely a beneficial mechanism for early development of educators, as this experience allows junior doctors to engage in a highly supported role as they navigate their first teaching experiences. The findings of positive attitudes towards medical education and desires to work in medical education are also likely partly explained by prior enjoyable teaching experience. However, it is likely that participation in the NPMT contributes to these attitudes and aspirations, as indicated by the respondent who continued engaging in near peer teaching, in the absence of a structured program, following NPMT involvement. These findings could be expanded through a further study to deeply explore tutors’ continuing teaching experience and the NPMT’s influence in this context.

The findings that respondents experienced enjoyment and perceived benefits from the program is not surprising given previously discussed benefits. Nonetheless, it is noteworthy that our respondents reported the strongest improvements in their communication skills and their ability to engage in feedback conversations after involvement with the NPMT. These qualities have been identified amongst the common attributes recognised as contributing to excellent clinical teaching alongside ‘good supervision skills’ and ‘organisational skills’ [36]. An interesting parallel can be drawn to an Italian study of anatomy tutors, where communication skills were ranked highest and technical skills were ranked last in self-perceived improvement, in alignment with our findings [11]. This may suggest that near-peer teaching may be more conducive to developing non-technical qualities rather than honing technical skills or clinical knowledge. This aspect could be further explored through qualitative longitudinal inquiry in order to provide a nuanced description of the tutors’ development through engagement in NPMT.

The main strength of this study is that it offers a novel insight into the experience of Australian junior doctors undertaking near-peer teaching in a teaching hospital network. However, there are also several limitations. A major limitation was a low overall response rate, which is likely largely explained by the low response rate from past tutors. This is, in large part, due to difficulty reaching past tutors as many have moved to different training contexts or obtained employment outside the health district. The vast majority of respondents in this survey were current participants and therefore these findings do not accurately reflect past tutors’ experiences, particularly as the program has grown and evolved over time. It is possible that tutors who dropped out of the program may have received less benefit from it and that these data may be subject to selection bias. Another major limitation is that respondents were restricted in describing their experience of the program by our survey structure – utilising visual analogue scales, with minimal ability to expand further on these themes. While we obtained valuable descriptions in the free text answer boxes, we were unable to delve deeper in the participants’ experience due to the anonymous nature of the survey. Ideally all survey responses would have been expanded upon in interview settings; however, this was not feasible in the current study format. Additionally, improvements reported by participants in this study are self-perceived and may not translate objectively. Finally, future research should explore other stakeholders’ perspectives including learners and patients, as these are key components of the program which are not explored here.

Despite the limitations, our study offers a practical example of how educational institutions can create structures to maintain emerging clinical educators’ motivation towards teaching. In particular, our findings show the effectiveness of a curating a collegiate environment and support resources in providing positive experiences in teaching. Integrating these within the framework recently proposed by Orsini and colleagues in their systematic review [37], we emphasise the importance of strategies that foster autonomous motivation.

## Conclusion

Junior doctors appear to benefit from engaging in near-peer programs in the Australian teaching hospital setting. Competing clinical priorities and inconvenient teaching times may impact tutor experience. Furthermore, this study indicates that junior doctors derived enjoyment and development of clinical skills from the program, which are important factors in increasing job satisfaction and ameliorating burn-out in this cohort. Participation appears to positively reinforce attitudes towards teaching and desires for future and continuing involvement in medical education. Prior near-peer teaching in medical school may explain ongoing enthusiasm for teaching in this cohort. Further research must include qualitative methodologies to more deeply explore the perspectives of Australian junior doctors’ regarding their teaching experiences to expand on these findings, as well as expand its scope to explore the perspectives of other major stakeholders including patients, students and supervisors.

## Supplementary Information

Additional file 1. NPMT Tutor Survey Instrument.

## Data Availability

The dataset analysed during the current study is available from the corresponding author on reasonable request.
