# Supplementary material for: Benefits of a near-peer program from the tutors’ perspective: a survey of Australian junior doctors in a regional teaching program
Source: BMC Med Educ. 2025 Feb 27;25:318. doi: 10.1186/s12909-025-06762-2 (PMC11866562; doi:10.1186/s12909-025-06762-2)
Supplement: Supplementary file 1 — Additional file 1. NPMT Tutor Survey Instrument. [file 12909_2025_6762_MOESM1_ESM.pdf]

NPMT Tutor Survey

Thank you very much for participating in our survey.

You will be asked about your general personal details and how you think participation in the NPMT program has impacted your ability, professional qualities, and your attitudes towards teaching while working and medical education.

This survey should take 10-15 minutes to complete.

NPMT Tutor Survey

Part 1: Tutor demographics

Have you participated in the NPMT program as a tutor?

- ○ Yes
- ○ No

What motivated you to participate in the NPMT program?

- ☐ Giving back to medical community
- ☐ Prior enjoyable teaching experience
- ☐ Adding to my CV
- ☐ Consolidation of own knowledge and skills
- ☐ Interest in working in medical education
- ☐ Other

Please detail your other motivation(s) for participating in the NPMT program.

____

When did you first participate in the NPMT program?	◯ 2022<br>◯ 2021<br>◯ 2020<br>◯ 2019<br>◯ 2018<br>◯ 2017<br>◯ 2016 or previously
Do you currently participate in the NPMT program?	◯ Yes<br>◯ No
When was the last time you participated in the NPMT program?	◯ 2022<br>◯ 2021<br>◯ 2020<br>◯ 2019<br>◯ 2018<br>◯ 2017<br>◯ 2016 or previously
How old are you?	__________________________________
What is your gender?	◯ Female<br>◯ Male<br>◯ Other<br>◯ Prefer not to say
Please specify your gender.	__________________________________
When you last participated in the NPMT program, what was your role in the hospital?	◯ Junior Medical Officer (Intern)<br>◯ Resident Medical Officer<br>◯ Senior Resident Medical Officer<br>◯ Registrar<br>◯ Career Medical Officer<br>◯ Fellow<br>◯ Consultant
When you last participated in the NPMT program, what was your postgraduate level of experience?	◯ PGY1<br>◯ PGY2<br>◯ PGY3<br>◯ PGY4<br>◯ PGY5<br>◯ PGY6 or above

Which medical school did you attend?	◯ Australian National University<br>◯ Bond University<br>◯ Charles Darwin University<br>◯ Charles Sturt University<br>◯ Curtin University<br>◯ Deakin University<br>◯ Flinders University<br>◯ Griffith University<br>◯ James Cook University<br>◯ Macquarie University<br>◯ Monash University<br>◯ University of Adelaide<br>◯ University of Melbourne<br>◯ University of Newcastle & University of New England<br>◯ University of New South Wales<br>◯ University of Notre Dame Fremantle<br>◯ University of Notre Dame Sydney<br>◯ University of Queensland<br>◯ University of Sydney<br>◯ University of Tasmania<br>◯ University of the Sunshine Coast<br>◯ University of Western Australia<br>◯ University of Wollongong<br>◯ Western Sydney University<br>◯ Overseas university
Did you participate in the NPMT program as a student?	◯ Yes<br>◯ No
Please name which medical school you attended (include country).	__________________________________
Prior to participating in the NPMT program, what previous teaching experience did you have as a tutor?	☐ Nil<br>☐ Bedside teaching<br>☐ Tutorials<br>☐ Skills sessions (practical, hands on teaching)<br>☐ Resource writing<br>☐ Workshop attendance<br>☐ Formal or mock examination<br>☐ Leadership role
Do you have a formal education qualification?	◯ Yes<br>◯ No
Please specify which qualification you have including the name of your degree, university and year of completion.	__________________________________
What areas of the NPMT program have you participated in?	☐ Bedside teaching<br>☐ Tutorials eg. Medicine, radiology<br>☐ Skills sessions<br>☐ Resource writing eg. tutorial, cheat sheets<br>☐ Workshop attendance<br>☐ Formal/mock examination<br>☐ Leadership role<br>☐ Other
Please specify which other area(s) of the NPMT program you have participated in.	__________________________________

What content areas/specialties of the NPMT program have you participated in?	☐ Medicine<br>☐ Surgery<br>☐ Critical Care (Emergency, ICU and/or Anaesthetics)<br>☐ Radiology<br>☐ Obstetrics and Gynaecology<br>☐ Paediatrics<br>☐ Psychiatry<br>☐ Other
Please specify the other content area/specialty of the NPMT program that you participated in.	__________________________________
On average, how often have you participated in the NPMT program?	◯ Once a week<br>◯ Once a fortnight<br>◯ Once a month<br>◯ Less than once a month

Part 2: Impacts of participating in the NPMT program.

Please answer these questions in context of your experience teaching within the NPMT program.

Do you believe that you have benefited from the program?

No benefit | Some benefit | Strong benefit

(Place a mark on the scale above)

Do you believe that your clinical knowledge has improved by teaching?

No improvement | Some improvement | Strong improvement

(Place a mark on the scale above)

Do you believe that your technical skills have improved by teaching?

No improvement | Some improvement | Strong improvement

(Place a mark on the scale above)

Do you believe that your communication skills have improved by teaching?

No improvement | Some improvement | Strong improvement

(Place a mark on the scale above)

Do you believe that your ability to give feedback has improved by teaching?

No improvement | Some improvement | Strong improvement

(Place a mark on the scale above)

Do you believe your organizational/planning skills have improved by teaching?

No improvement | Some improvement | Strong improvement

(Place a mark on the scale above)

Do you believe your own ability to learn has improved by teaching?

No improvement | Some improvement | Strong improvement

(Place a mark on the scale above)

Do you feel more confident as a clinician by teaching?

No more confident | Somewhat more confident | Strongly more confident

(Place a mark on the scale above)

Do you feel more confident as a clinical teacher?

No more confident | Somewhat more confident | Strongly more confident

(Place a mark on the scale above)

Part 3: Teaching within the work environment

Did you enjoy teaching with the NPMT program?

Did not enjoy at all | Somewhat enjoyed | Strongly enjoyed

(Place a mark on the scale above)

Were you able to teach at times convenient to you?

Never convenient | Sometimes convenient | Always convenient

(Place a mark on the scale above)

Prior to participating in NPMT, did you find teaching stressful?

Minimally stressful | Sometimes stressful | Majorly stressful

(Place a mark on the scale above)

Do you believe participating in the NPMT program relieved stress associated with teaching?

No relief of stress | Some relief of stress | Strong relief of stress

(Place a mark on the scale above)

What aspects of the NPMT program helped relieve stress associated with teaching?

- ☐ Nil
- ☐ NPMT website and resources
- ☐ Teaching workshop
- ☐ Collegiate environment
- ☐ Logistical support eg. giving your pager to others
- ☐ Other

Please detail how the NPMT program helped relieve stress.

____

Were you able to teach during allocated work shifts?

- ◯ Yes
- ◯ No

Were you able to teach in hours, out of hours or both?

- ◯ In hours
- ◯ Out of hours
- ◯ Both

How much teaching was done in hours vs out of hours?

Mostly in hours | Evenly mixed | Mostly out of hours

(Place a mark on the scale above)

Were you able to teach without interruptions from work responsibilities?

No, never | Sometimes | Yes, always

(Place a mark on the scale above)

Were you able to leave your pager with a colleague when teaching?

No, never | Sometimes | Yes, always

(Place a mark on the scale above)

Part 4: Attitudes towards teaching and medical education

Would you recommend the NPMT program to yourself if you were a medical student at the Central Coast Clinical School?

Would not recommend at all — Neutral — Would strongly recommend

(Place a mark on the scale above)

Do you believe that every doctor should participate in medical student teaching?

No, not at all — Neutral — Yes, strongly so

(Place a mark on the scale above)

Would you like to improve your teaching skills?

No, not at all — Neutral — Yes, strongly so

(Place a mark on the scale above)

How useful did you find the NPMT website npmteaching.com?

Not at all useful — Somewhat useful — Strongly useful

(Place a mark on the scale above)

Do you believe you will make teaching a major part of your career?

No, not at all likely — Neutral — Yes, strongly likely

(Place a mark on the scale above)

Do you believe that you will pursue more formal teaching opportunities in the future?

No, not at all likely — Neutral — Yes, strongly likely

(Place a mark on the scale above)

Do you believe your experience with the NPMT program has influenced your desire to pursue future formal teaching opportunities?

No, not at all — Neutral — Yes, strongly so

(Place a mark on the scale above)

Do you participate in any formal teaching activities?

○ Yes ○ No

On average, how often do you participate in formal teaching activities?

○ Once a week
○ Once a fortnight
○ Once a month
○ Less than once a month

Do you believe that your experience with the NPMT program influenced your decision to continue to teach?

No, not at all — Neutral — Yes, strongly so

(Place a mark on the scale above)

Do you still access the website npmteaching.com?

○ Yes ○ No

Do you believe that participating in the NPMT program has helped progress your career?

No, not at all — Neutral — Yes, strongly so

(Place a mark on the scale above)

Do you have anything that you would like to say about your experience with the NPMT?
